# Comparison of Omron and ActiGraph monitors in estimating daily step counts and time spent in moderate to vigorous physical activity in free-living adults

**DOI:** 10.1136/bmjsem-2024-002402

**Published:** 2025-03-06

**Authors:** Hiroko Shimura, Shinpei Okada, Kazushi Maruo, Kaori Daimaru, Naoki Deguchi, Yoshinori Fujiwara, Hiroyuki Sasai

**Affiliations:** 1 Research Team for Promoting Independence and Mental Health, Tokyo Metropolitan Institute for Geriatrics and Gerontology, Tokyo, Japan; 2 Physical Education and Medicine Research Foundation, Nagano, Japan; 3 Department of Preventive Medicine and Public Health, Tokyo Medical University, Tokyo, Japan; 4 Department of Biostatistics, Institute of Medicine, University of Tsukuba, Ibaraki, Japan; 5 Tokyo Metropolitan Institute for Geriatrics and Gerontology, Tokyo, Japan

**Keywords:** Physical activity, Measurement, Accelerometer

## Abstract

**Objective:**

This study compared the Omron Active style Pro HJA-750C (OM) and the ActiGraph wGT3X-BT (AG) in estimating daily physical activity—step counts and time spent in moderate to vigorous physical activity (MVPA)—in free-living adults.

**Methods:**

Japanese adults without gait abnormalities wore both devices during waking hours for seven consecutive days. Data were aggregated into daily steps and MVPA. A valid day required ≥10 hours of AG wear time with ≥100 and <50 000 accumulated steps from both devices. Agreement was assessed using Bland-Altman plots with multilevel analysis.

**Results:**

The final dataset included 129 participants (age 23–89 years, 50.4% women), totalling 887 observations (5–7 daily observations/participant). OM estimated an overall mean of 7456 (SE 253) steps/day and 68.9 (SE 2.8) min/day in MVPA. Bland-Altman plots showed that OM estimated −56 steps/day (95% limits of agreement (LoA) = −1599; 1486) and +23 min/day (LoA = −17; 63) in MVPA compared with AG. Differences tended to increase with higher mean estimates for both step counts and MVPA.

**Conclusion:**

OM estimated substantially more daily time spent in MVPA but showed similar daily step counts compared with AG. Differences were larger with higher activity levels.

**WHAT IS ALREADY KNOWN ON THIS TOPIC**

- The previous generation of the Omron activity monitor, the HJA-350IT, tended to estimate more daily time spent in moderate to vigorous physical activity (MVPA) while providing similar daily step counts compared with the ActiGraph monitor. However, it remains unclear whether these findings apply to the latest generation Omron activity monitor.

**WHAT THIS STUDY ADDS**

- Comparing the Omron Active style Pro HJA-750C (OM) and the ActiGraph wGT3X-BT (AG), this study found that OM estimated more daily time spent in MVPA, but similar daily step counts compared with AG in free-living adults. Proportional bias was observed for both MVPA and step counts. These findings were analysed using Bland-Altman plots with multilevel analysis.

**HOW THIS STUDY MIGHT AFFECT RESEARCH, PRACTICE OR POLICY**

- Researchers and practitioners should be aware of OM’s tendency to estimate more time spent in MVPA compared with AG.

## Introduction

 Reliable measures of physical activity are essential for research and designing interventions targeting adult physical activity. Research-grade activity monitors provide objective estimates of step counts and physical activity intensity through time series data, with daily accumulation of step counts and time spent in moderate to vigorous physical activity (MVPA) capturing individual day-to-day variations in overall physical activity.

In Japan, the Omron and ActiGraph monitors are popular in physical activity research. Although the ActiGraph accounts for approximately 50% of studies on physical activity assessment worldwide,^1^ the Omron is commonly preferred in Japan due to its cost-effectiveness, particularly in large-scale studies.^2 3^ The latest generations of these devices, ActiGraph’s wGT3X-BT (ActiGraph LLC, Pensacola, Florida, USA) and Omron’s Active style Pro HJA-750C (Omron Healthcare Co., Kyoto, Japan) cost approximately JPY 60 000 and 20 000, respectively (as of October 2024).

Understanding the comparability between different monitors is essential for accurate data interpretation across studies and populations. Although some studies have compared earlier Omron and ActiGraph generations, such as the HJA-350IT and GT3X+, under free-living conditions,^4 5^ comparisons between the latest generations are lacking. The HJA-350IT’s validity has been established in controlled laboratory settings,^6 7^ and the HJA-750C, which is smaller than the HJA-350IT, uses the same acceleration sensor and algorithms.^8^ However, it was reported that the HJA-750C underestimated daily time spent in MVPA compared with the HJA-350IT, with no significant difference in daily step counts under free-living settings.^9^ Furthermore, a lab-based study showed that acceleration output from GT3X+ and wGT3X-BT differed at the highest frequency.^10^ These reports suggest that previous findings^4 5^ may not fully apply when comparing the HJA-750C and wGT3X-BT.

This study compared the daily estimates of step counts and time spent in MVPA, derived from the HJA-750C (hereafter OM) and wGT3X-BT (hereafter AG) in adults under free-living conditions.

## Methods

### Participants

A convenience sample of adults aged 20–89 years without gait abnormalities was recruited from communities across several prefectures in Japan, including the National Capital Region (Tokyo, Kanagawa, Saitama and Yamanashi) and other prefectures (Nagano and Gifu).

### Data collection

#### Baseline characteristics

Demographic and anthropometric information was collected during the introductory session (day 0). Participants self-reported their age, gender, living arrangement, employment status and perceived health. Body mass index (BMI) was calculated from self-reported height and weight and classified as normal (<25 kg/m^2^) or overweight/obese (≥25 kg/m^2^).

#### Physical activity in free-living conditions

Physical activity data were collected for seven consecutive days (days 1–7). Participants were instructed to wear an elastic belt around their waist, placing an OM device (size: 52 mm × 40 mm × 12 mm; weight: 23 g) on the right side and an AG device (size: 46 mm × 33 mm × 15 mm; weight: 19 g) on the left, during their waking hours, except when engaging in water-based activities (eg, showering and swimming) or high-contact sports. The devices were returned using a pre-paid envelope on day 8.

The data from the OM and AG devices were downloaded using dedicated software provided by Omron (V.2.9; Omron Healthcare Co.) and ActiLife software (V.6.13.4; ActiGraph LLC), respectively. To synchronise the time settings, all devices were initialised on the same computer before data collection.

This study analysed minute-by-minute data (ie, 1-min epoch data) on activity counts and step counts from the AG (sampled at 50 Hz), along with minute-by-minute metabolic equivalents (METs) and day-by-day data (ie, 1-day epoch data) on step count from the OM (sampled at 32 Hz).^8^ These data were provided by the aforementioned software. The OM did not provide step count data on a minute-by-minute basis due to proprietary algorithms.

### Activity monitor data processing

For step counts, minute-by-minute AG data were aggregated into daily values and merged with day-by-day OM data using participant ID and timestamp as keys. Because OM step count data could not be excluded minute-by-minute, both OM and AG data were retained to avoid significant data loss, even if the period was labelled as non-wear by the AG.^11 12^

For MVPA, the minute-by-minute OM and AG data were merged using the aforementioned keys. Data were then aggregated into daily time spent in MVPA (OM: ≥3.0 METs; AG: ≥2690 vector magnitude counts per minute for the AG^13^) for each participant after excluding minute-by-minute observations classified as non-wear by the AG.^11 12^ Because the OM was attached to the same belt as the AG, non-wear periods identified by the AG were also applied to the OM.

A valid day was defined as having ≥10 hours/day of AG wear time (convention for compliant wear time),^14^ with an accumulation of ≥100 and <50 000 steps/day (to exclude outliers)^15^ from both AG and OM.

### Statistical analysis

Participant characteristics were summarised as means and SD, or as frequencies and percentages. To assess the impact of including non-wear periods on step counts, we examined the distribution of AG’s minute-by-minute step counts during non-wear periods. A multilevel model, including intercept only, was used to estimate the overall mean and SE of step counts and time spent in MVPA, accounting for the dataset’s hierarchical structure (maximum seven valid daily observations per participant). Bland-Altman plots^16^ were used to visualise agreement between OM and AG for each measure across each day. A multilevel model, including intercept only, was also used to estimate the overall mean of the paired difference between the devices and 95% limits of agreement (LoA).^17^,^19^ Proportional bias was quantified based on the slope parameter of the multilevel model, including the average between the devices for each outcome as a fixed effect. Statistical analyses were performed using SPSS 29.0.1.0 (IBM Corporation, Armonk, New York, USA) and R V.4.4.0. The lme4 package in R was used for multilevel modelling.^20^

## Results

All 130 participants enrolled in the study completed data collection; however, one participant was excluded due to an unexpected OM malfunction. The final sample included 129 participants (age 23–89 years, 50.4% women); their characteristics are presented in table 1.

**Table 1:** Descriptive characteristics of the participants

	All (n=129)	Men (n=64)	Women (n=65)
Age	53.5 (18.9)	52.8 (18.8)	54.2 (19.1)
Age group			
20–29	16 (12.4%)	6 (9.4%)	10 (15.4%)
30–39	21 (16.3%)	14 (21.9%)	7 (10.8%)
40–49	20 (15.5%)	10 (15.6%)	10 (15.4%)
50–59	23 (17.8%)	13 (20.3%)	10 (15.4%)
60–69	14 (10.9%)	4 (6.3%)	10 (15.4%)
70–79	22 (17.1%)	9 (14.1%)	13 (20.0%)
80–89	13 (10.1%)	8 (12.5%)	5 (7.7%)
Living area			
National Capital Region	59 (45.7%)	27 (42.9%)	31 (47.7%)
Other prefectures	70 (54.3%)	36 (57.1%)	34 (52.3%)
Living arrangement			
Living alone	36 (27.9%)	11 (17.2%)	25 (38.5%)
Not living alone	93 (72.1%)	53 (82.8%)	40 (61.5%)
Employment status			
Work full-time	80 (62.0%)	45 (70.3%)	35 (53.8%)
Work part-time	20 (15.5%)	7 (10.9%)	13 (20.0%)
Not working	29 (22.5%)	12 (18.8%)	17 (26.2%)
Perceived health			
Excellent	22 (17.1%)	12 (18.8%)	10 (15.4%)
Very good	55 (42.6%)	27 (42.2%)	28 (43.1%)
Good	52 (40.3%)	25 (39.1%)	27 (41.5%)
Fair or Poor	0 (0%)	0 (0%)	0 (0%)
Height (cm)	163.4 (9.3)	171.2 (5.1)	155.7 (5.3)
Weight (kg)	59.8 (11.5)	67.7 (9.1)	52.1 (7.7)
BMI (kg/m^2^)	22.3 (3.2)	23.1 (3.0)	21.5 (3.2)
Overweight/Obese (BMI≥25 kg/m^2^)	24 (18.6%)	14 (21.9%)	10 (15.4%)

Note. The table presents the mean (SD) for continuous variables and n (%) for categorical variables..

BMI, body mass index

A total of 887 daily observations were analysed, with each participant providing 5–7 daily observations. For step count data of AG, the maximum value during non-wear periods was 25 steps per minute, with 99.98% of data showing 0 steps per minute. The estimated overall means for OM were 7456 (SE 253) steps per day and 68.9 (SE 2.8) minutes per day in MVPA, and for AG were 7512 (SE 243) steps per day and 45.7 (SE 2.2) minutes per day in MVPA.

**Figure 1:**
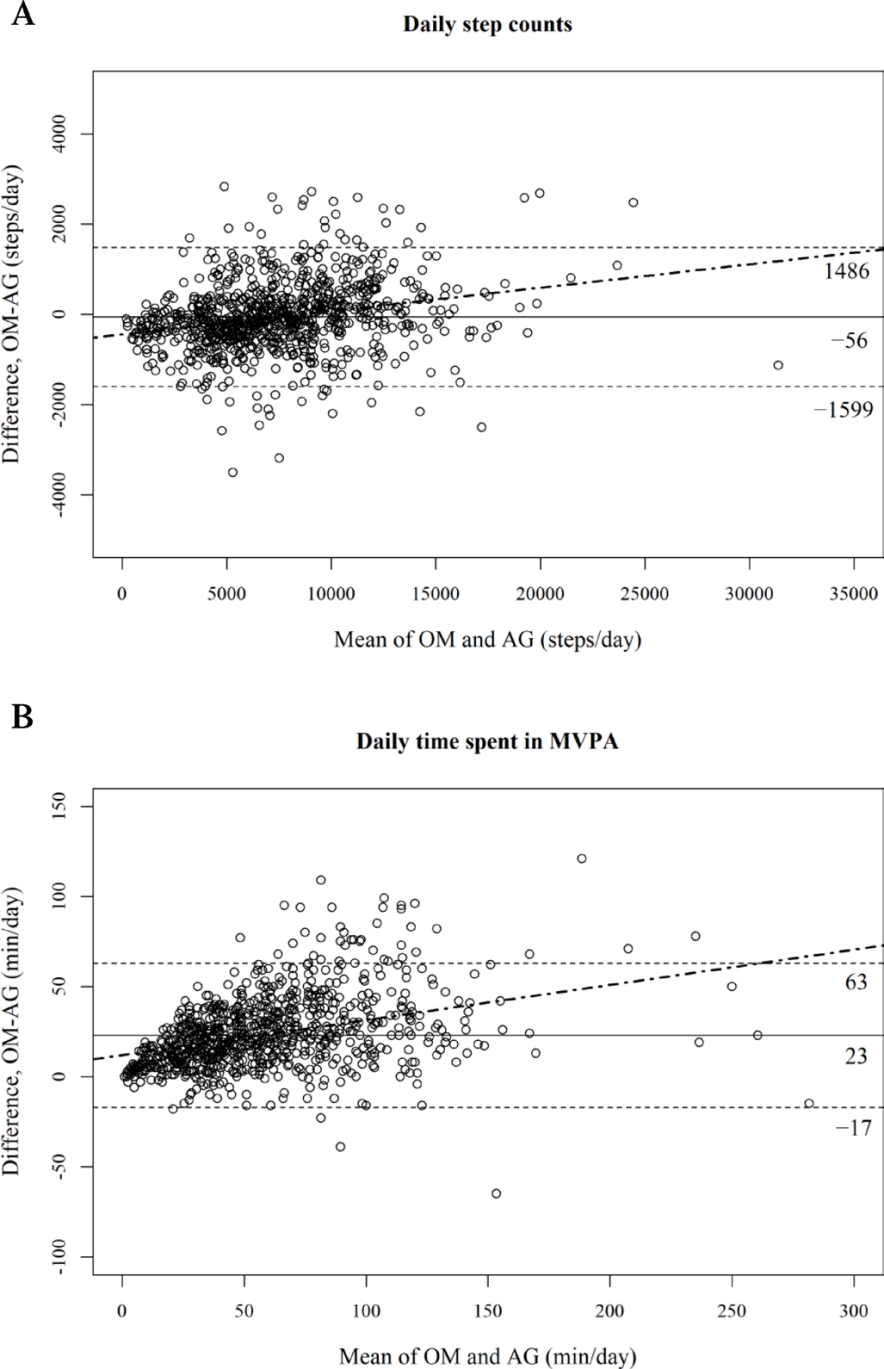
Bland-Altman plot comparing the Omron HJA-750C (OM) with the ActiGraph wGT3X-BT (AG) for daily estimates of physical activity (887 observations of 129 participants). The solid lines represent the mean difference, the dashed lines represent the 95% limits of agreement and the dot-dash lines represent the linear regression lines by multilevel model. MVPA, moderate to vigorous physical activity.

Figure 1 shows the Bland-Altman plots comparing the OM and AG estimates. OM estimated 56 fewer steps per day (LoA = −1599 to 1486; figure 1A) and 23 more minutes per day in MVPA (LoA = −17 to 63; figure 1B) compared with those estimated by the AG. Also, for both step counts and MVPA, the difference tended to increase as the mean estimates increased, with significant regression slopes observed for step counts (0.052, SE 0.006, p<0.001) and MVPA (0.195, SE 0.016, p<0.001).

## Discussion

### Principal findings

This study conducted in adults under free-living conditions found that the OM and AG provided generally similar daily estimates of step counts, differing by only 56 steps per day, roughly equivalent to 0.6 min of walking per day (assuming 100 steps per minute^21^). However, OM estimated substantially more MVPA compared with AG, with a mean difference of 23 min per day or about 161 min per week—exceeding the recommended 150 min of MVPA per week.^22^ Results also suggested proportional bias for both MVPA and step counts, with differences increasing at higher mean estimates, though this bias was more pronounced for MVPA than for step counts.

### Comparison with previous studies

An earlier study reported that the HJA-350 IT (the predecessor to the OM) estimated 318 more steps per day and 28.2 additional MVPA minutes per day than the GT3X+ (the predecessor to the AG) based on 2 days of data from 50 working adults.^5^ Another 7-day study using these devices found that the average daily steps count was approximately the same for both younger and older adults.^4^ Our findings largely align with those studies,^4 5^ despite using updated generations of Omron and ActiGraph monitors. The observed differences in the magnitude of fixed biases likely stem from participant characteristics, measurement periods and analytical approaches such as multilevel modelling. For MVPA, fixed and proportional biases may result from the OM’s algorithm, which applies three different MET calculation equations based on activity type (ie, sedentary, household or locomotive),^6 7^ whereas AG estimates METs from count values alone, regardless of activity type.^13^ Step detection, which relies on simple peak detection from acceleration data, may show fewer differences between manufacturers compared with MVPA as the latter likely involves more complex processing.

### Strengths and limitations

The strengths of this study include a large, diverse sample, free-living conditions enhancing ecological validity,^23^ and the use of multilevel analysis to handle repeated measures, thereby improving agreement estimate accuracy.^17^ Few studies have employed multilevel analysis despite its suitability for hierarchical datasets.^18 24^

A limitation is that non-wear periods were not excluded from the step count analysis; while most AG step counts during these periods were likely zero with minimal impact on daily totals, this could not be confirmed for OM due to the lack of minute-by-minute data. Additionally, the AG cut-point used in this study,^13^ developed from treadmill walking data without considering lifestyle activities, may affect its accuracy for true MVPA, with the higher MVPA estimates by OM potentially reflecting algorithmic differences rather than inaccuracies. Furthermore, due to the hierarchical structure of the data, ordinary least products regression could not be applied, and proportional bias was quantified using multilevel linear regression instead.

## Conclusion

In free-living adults, the OM estimated substantially more daily time spent in MVPA but provided comparable daily step counts relative to the AG.
